# 2-(3-Pyridinio)benzimidazolium penta­chloridoanti­monate(III) monohydrate

**DOI:** 10.1107/S1600536809018935

**Published:** 2009-05-23

**Authors:** Li-Jing Cui, Hai-Jun Xu, Ke-Ji Pan

**Affiliations:** a Ordered Matter Science Research Center, College of Chemistry and Chemical Engineering, Southeast University, Nanjing 211189, People’s Republic of China

## Abstract

In the title compound, (C_12_H_11_N_3_)[SbCl_5_]·H_2_O, the Sb^III^ centre is surrounded by five Cl atoms and displays a distorted square-pyramidal coordination geometry. The dihedral angle formed by the plane of the imidazole ring system with the pyridine ring is 4.380 (15)°. The crystal structure is stabilized by N—H⋯Cl, O—H⋯Cl and N—H⋯O hydrogen bonds, forming a three-dimensional network.

## Related literature

For the pharmacologic activity of benzimidazole derivatives, see: Minoura *et al.* (2004 ▶); Pawar *et al.* (2004 ▶); Demirayak *et al.* (2002 ▶).
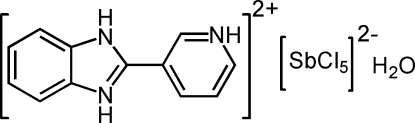


## Experimental

### 

#### Crystal data

(C_12_H_11_N_3_)[SbCl_5_]·H_2_O
*M* *_r_* = 514.25
Monoclinic, 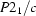

*a* = 9.2619 (19) Å
*b* = 13.425 (3) Å
*c* = 14.380 (3) Å
β = 102.27 (3)°
*V* = 1747.2 (7) Å^3^
*Z* = 4
Mo *K*α radiation
μ = 2.35 mm^−1^
*T* = 293 K
0.25 × 0.22 × 0.19 mm

#### Data collection

Rigaku SCXmini diffractometer
Absorption correction: multi-scan (*CrystalClear*; Rigaku, 2005 ▶) *T*_min_ = 0.892, *T*_max_ = 0.964 (expected range = 0.592–0.640)
15623 measured reflections
3410 independent reflections
3037 reflections with *I* > 2σ(*I*)
*R*_int_ = 0.043

#### Refinement

*R*[*F* ^2^ > 2σ(*F* ^2^)] = 0.028
*wR*(*F* ^2^) = 0.068
*S* = 0.93
3410 reflections
199 parameters
6 restraints
H-atom parameters constrained
Δρ_max_ = 0.32 e Å^−3^
Δρ_min_ = −0.48 e Å^−3^

### 

Data collection: *CrystalClear* (Rigaku, 2005 ▶); cell refinement: *CrystalClear*; data reduction: *CrystalClear*; program(s) used to solve structure: *SHELXS97* (Sheldrick, 2008 ▶); program(s) used to refine structure: *SHELXL97* (Sheldrick, 2008 ▶); molecular graphics: *SHELXTL* (Sheldrick, 2008 ▶); software used to prepare material for publication: *SHELXL97*.

## Supplementary Material

Crystal structure: contains datablocks I, global. DOI: 10.1107/S1600536809018935/rz2324sup1.cif

Structure factors: contains datablocks I. DOI: 10.1107/S1600536809018935/rz2324Isup2.hkl

Additional supplementary materials:  crystallographic information; 3D view; checkCIF report

## Figures and Tables

**Table 1:** Hydrogen-bond geometry (Å, °)

*D*—H⋯*A*	*D*—H	H⋯*A*	*D*⋯*A*	*D*—H⋯*A*
N1—H1*A*⋯O1*W*	0.86	1.82	2.652 (4)	161
N3—H3*B*⋯Cl5^i^	0.86	2.28	3.056 (3)	150
N2—H2*B*⋯Cl2^ii^	0.86	2.48	3.179 (3)	139
O1*W*—H1*WA*⋯Cl2^iii^	0.85	2.35	3.197 (3)	172
O1*W*—H1*WB*⋯Cl3^iv^	0.85	2.35	3.198 (3)	174

Symmetry codes: (i) 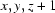; (ii) 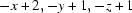; (iii) 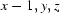; (iv) 
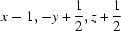
.

**Fig. 1.**
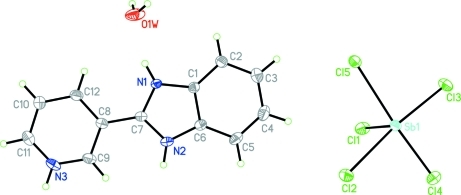
The molecular structure of the title compound, showing the atomic numbering scheme. Displacement ellipsoids are drawn at the 30% probability level.

**Fig. 2.**
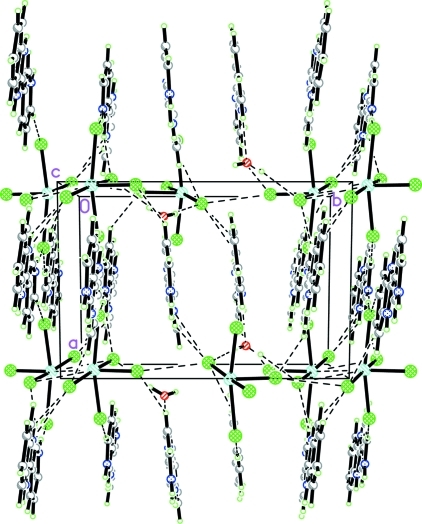
Packing diagram of the title compound, showing the structure along the c axis. Intermolecular H bonds are shown as dashed lines.

## supplementary crystallographic information

### Comment

Benzimidazole and its derivatives have received great attention owing to their pharmacologic activities, such as antidiabetic (Minoura *et al.*, 2004), antifungal (Pawar *et al.*,2004), and anticancer (Demirayak *et al.*, 2002) activities. In this paper, the crystal structure of the title compound is reported.

The asymmetric unit of the title compound (Fig. 1) contains a 2-(3'-pyridinio)benzimidazolium dication, a pentachloroantimonate dianion and a water molecule. In the anion, the antimony(III) atom is coordinated by five chloride anions in a distorted square-pyramidal geometry. The Sb—Cl distances are in the range 2.3687 (10)- 2.7522 (11) Å. In the cation, the pyridine ring and the imidazole ring system are nearly coplanar, the dihedral angle they form being 4.360 (15)°. The crystal packing (Fig. 2) is stabilized by intermolecular N—H···O, N—H···C and O—H···Cl hydrogen bonds (Table 1), resulting in the formation of a three-dimensional network.

### Experimental

To a mixture of 2-(3'-pyridyl)benzimidazole (0.1 mmol) and water (7 ml), concentrated hydrochloric acid (12 M) was added dropwise till complete dissolution of the solid phase. Concentrated hydrochloric acid was similarly added dropwise to dissolve the solid phase persisting in a mixture of antimony trichloride (0.3 mmol) and water (7 ml). The two solutions were then mixed and stirred for 20 minutes. The resulting precipitate was filtered off and dissolved in hydrochloric acid. Colourless crystals suitable for X-ray analysis were formed after several weeks on slow evaporation of the solvent at room temperature.

### Refinement

H atoms bound to C and N atoms were positioned geometrically and treated as riding, with C—H = 0.93 Å, N—H = 0.86 Å, and with *U*_iso_(H) = 1.2U_eq_(C, N). Water H atoms were located in a difference Fourier map and refined with O—H = 0.85 Å and with *U*_iso_(H) = 1.2U_eq_(O).

### Crystal data

**Table:**

(C_12_H_11_N_3_)[SbCl_5_]·H_2_O	*F*(000) = 1000
*M**_r_* = 514.25	*D*_x_ = 1.955 Mg m^−^^3^
Monoclinic, *P*2_1_/*c*	Mo *K*α radiation, λ = 0.71073 Å
Hall symbol: -P 2ybc	Cell parameters from 1647 reflections
*a* = 9.2619 (19) Å	θ = 3.0–27.6°
*b* = 13.425 (3) Å	µ = 2.35 mm^−^^1^
*c* = 14.380 (3) Å	*T* = 293 K
β = 102.27 (3)°	Prism, colourless
*V* = 1747.2 (7) Å^3^	0.25 × 0.22 × 0.19 mm
*Z* = 4	

### Data collection

**Table:**

Rigaku SCXmini diffractometer	3410 independent reflections
Radiation source: fine-focus sealed tube	3037 reflections with *I* > 2σ(*I*)
graphite	*R*_int_ = 0.043
Detector resolution: 13.6612 pixels mm^-1^	θ_max_ = 26.0°, θ_min_ = 3.0°
ω scans	*h* = −11→11
Absorption correction: multi-scan (*CrystalClear*; Rigaku, 2005)	*k* = −16→16
*T*_min_ = 0.892, *T*_max_ = 0.964	*l* = −17→17
15623 measured reflections	

### Refinement

**Table:**

Refinement on *F*^2^	Primary atom site location: structure-invariant direct methods
Least-squares matrix: full	Secondary atom site location: difference Fourier map
*R*[*F*^2^ > 2σ(*F*^2^)] = 0.028	Hydrogen site location: inferred from neighbouring sites
*wR*(*F*^2^) = 0.068	H-atom parameters constrained
*S* = 0.93	*w* = 1/[σ^2^(*F*_o_^2^) + (0.0319*P*)^2^ + 2.5497*P*] where *P* = (*F*_o_^2^ + 2*F*_c_^2^)/3
3410 reflections	(Δ/σ)_max_ = 0.001
199 parameters	Δρ_max_ = 0.32 e Å^−^^3^
6 restraints	Δρ_min_ = −0.47 e Å^−^^3^

### Special details

**Table:**

Geometry. All e.s.d.'s (except the e.s.d. in the dihedral angle between two l.s. planes) are estimated using the full covariance matrix. The cell e.s.d.'s are taken into account individually in the estimation of e.s.d.'s in distances, angles and torsion angles; correlations between e.s.d.'s in cell parameters are only used when they are defined by crystal symmetry. An approximate (isotropic) treatment of cell e.s.d.'s is used for estimating e.s.d.'s involving l.s. planes.
Refinement. Refinement of *F*^2^ against ALL reflections. The weighted *R*-factor *wR* and goodness of fit *S* are based on *F*^2^, conventional *R*-factors *R* are based on *F*, with *F* set to zero for negative *F*^2^. The threshold expression of *F*^2^ > σ(*F*^2^) is used only for calculating *R*-factors(gt) *etc*. and is not relevant to the choice of reflections for refinement. *R*-factors based on *F*^2^ are statistically about twice as large as those based on *F*, and *R*- factors based on ALL data will be even larger.

### Fractional atomic coordinates and isotropic or equivalent isotropic displacement parameters (Å^2^)

**Table:**

	*x*	*y*	*z*	*U*_iso_*/*U*_eq_	
Sb1	0.98691 (2)	0.410246 (16)	0.112742 (15)	0.02964 (9)	
Cl1	0.98813 (10)	0.24807 (7)	0.17778 (7)	0.0451 (2)	
Cl2	1.06091 (10)	0.48665 (7)	0.29163 (6)	0.0446 (2)	
Cl3	0.93983 (10)	0.34774 (8)	−0.06139 (6)	0.0445 (2)	
Cl4	1.25689 (10)	0.38926 (8)	0.11321 (8)	0.0516 (3)	
Cl5	0.68651 (10)	0.39170 (8)	0.09585 (6)	0.0434 (2)	
C1	0.5091 (3)	0.3717 (2)	0.4685 (2)	0.0280 (7)	
C2	0.4622 (4)	0.3711 (3)	0.3703 (2)	0.0346 (7)	
H2A	0.3631	0.3642	0.3409	0.042*	
C3	0.5715 (4)	0.3813 (3)	0.3187 (3)	0.0412 (9)	
H3A	0.5457	0.3804	0.2526	0.049*	
C4	0.7199 (4)	0.3929 (3)	0.3638 (3)	0.0463 (10)	
H4A	0.7901	0.3989	0.3265	0.056*	
C5	0.7658 (4)	0.3958 (3)	0.4611 (3)	0.0433 (9)	
H5A	0.8646	0.4046	0.4905	0.052*	
C6	0.6566 (4)	0.3849 (2)	0.5131 (2)	0.0304 (7)	
C7	0.5252 (3)	0.3714 (2)	0.6244 (2)	0.0280 (7)	
C8	0.4838 (4)	0.3694 (2)	0.7169 (2)	0.0289 (7)	
C9	0.5913 (4)	0.3773 (3)	0.7992 (2)	0.0376 (8)	
H9A	0.6906	0.3812	0.7963	0.045*	
N3	0.5509 (4)	0.3794 (2)	0.8830 (2)	0.0421 (7)	
H3B	0.6190	0.3841	0.9338	0.051*	
C11	0.4115 (5)	0.3745 (3)	0.8920 (3)	0.0417 (9)	
H11A	0.3891	0.3776	0.9520	0.050*	
C12	0.3373 (4)	0.3627 (3)	0.7250 (2)	0.0364 (8)	
H12A	0.2630	0.3566	0.6705	0.044*	
N1	0.4320 (3)	0.3631 (2)	0.54084 (18)	0.0285 (6)	
H1A	0.3383	0.3538	0.5328	0.034*	
N2	0.6611 (3)	0.3840 (2)	0.6097 (2)	0.0335 (6)	
H2B	0.7396	0.3905	0.6536	0.040*	
C10	0.3011 (4)	0.3649 (3)	0.8128 (3)	0.0407 (8)	
H10A	0.2031	0.3599	0.8182	0.049*	
O1W	0.1429 (3)	0.3470 (2)	0.4754 (2)	0.0636 (9)	
H1WA	0.1260	0.3892	0.4303	0.076*	
H1WB	0.0951	0.2925	0.4676	0.076*	

### Atomic displacement parameters (Å^2^)

**Table:**

	*U*^11^	*U*^22^	*U*^33^	*U*^12^	*U*^13^	*U*^23^
Sb1	0.02958 (13)	0.02996 (14)	0.02782 (13)	−0.00150 (9)	0.00262 (9)	0.00183 (9)
Cl1	0.0391 (5)	0.0362 (5)	0.0563 (6)	−0.0002 (4)	0.0014 (4)	0.0136 (4)
Cl2	0.0442 (5)	0.0505 (5)	0.0366 (5)	−0.0094 (4)	0.0033 (4)	−0.0069 (4)
Cl3	0.0465 (5)	0.0524 (6)	0.0302 (4)	0.0073 (4)	−0.0016 (4)	−0.0029 (4)
Cl4	0.0338 (5)	0.0687 (7)	0.0536 (6)	−0.0061 (4)	0.0121 (4)	−0.0013 (5)
Cl5	0.0362 (5)	0.0638 (6)	0.0294 (4)	0.0076 (4)	0.0051 (4)	−0.0009 (4)
C1	0.0268 (16)	0.0268 (16)	0.0303 (17)	−0.0023 (13)	0.0056 (13)	0.0006 (13)
C2	0.0346 (18)	0.0373 (19)	0.0307 (17)	−0.0029 (15)	0.0042 (14)	0.0015 (15)
C3	0.050 (2)	0.041 (2)	0.0347 (19)	0.0018 (17)	0.0125 (17)	0.0028 (16)
C4	0.043 (2)	0.052 (2)	0.050 (2)	0.0057 (18)	0.0234 (19)	0.0063 (18)
C5	0.0252 (18)	0.054 (2)	0.052 (2)	0.0025 (16)	0.0107 (16)	−0.0012 (18)
C6	0.0258 (16)	0.0304 (17)	0.0345 (18)	0.0018 (13)	0.0051 (14)	0.0005 (14)
C7	0.0243 (16)	0.0278 (16)	0.0296 (17)	−0.0012 (13)	0.0003 (13)	0.0004 (13)
C8	0.0297 (16)	0.0262 (16)	0.0284 (16)	−0.0001 (13)	0.0011 (13)	0.0001 (13)
C9	0.0345 (19)	0.042 (2)	0.0316 (18)	−0.0054 (15)	−0.0032 (15)	−0.0015 (15)
N3	0.0470 (19)	0.0465 (19)	0.0260 (15)	−0.0043 (14)	−0.0073 (13)	−0.0020 (13)
C11	0.056 (2)	0.039 (2)	0.0301 (19)	−0.0013 (17)	0.0093 (17)	−0.0027 (16)
C12	0.0316 (18)	0.043 (2)	0.0312 (18)	−0.0028 (15)	−0.0005 (14)	0.0018 (15)
N1	0.0199 (13)	0.0392 (16)	0.0252 (13)	−0.0065 (11)	0.0026 (10)	0.0025 (12)
N2	0.0215 (13)	0.0430 (17)	0.0331 (15)	−0.0011 (12)	−0.0008 (11)	−0.0007 (13)
C10	0.039 (2)	0.048 (2)	0.0365 (19)	−0.0003 (17)	0.0103 (16)	−0.0029 (17)
O1W	0.0352 (15)	0.074 (2)	0.073 (2)	−0.0205 (14)	−0.0072 (14)	0.0269 (17)

### Geometric parameters (Å, °)

**Table:**

Sb1—Cl1	2.3687 (10)	C7—N1	1.327 (4)
Sb1—Cl4	2.5149 (11)	C7—C8	1.461 (4)
Sb1—Cl3	2.5885 (10)	C8—C9	1.379 (5)
Sb1—Cl2	2.7184 (11)	C8—C12	1.389 (5)
Sb1—Cl5	2.7522 (11)	C9—N3	1.336 (5)
C1—C2	1.386 (5)	C9—H9A	0.9300
C1—N1	1.387 (4)	N3—C11	1.327 (5)
C1—C6	1.391 (4)	N3—H3B	0.8600
C2—C3	1.383 (5)	C11—C10	1.365 (5)
C2—H2A	0.9300	C11—H11A	0.9300
C3—C4	1.398 (6)	C12—C10	1.374 (5)
C3—H3A	0.9300	C12—H12A	0.9300
C4—C5	1.374 (6)	N1—H1A	0.8600
C4—H4A	0.9300	N2—H2B	0.8600
C5—C6	1.388 (5)	C10—H10A	0.9300
C5—H5A	0.9300	O1W—H1WA	0.8501
C6—N2	1.381 (4)	O1W—H1WB	0.8499
C7—N2	1.331 (4)		
			
Cl1—Sb1—Cl4	88.55 (4)	N2—C7—N1	108.8 (3)
Cl1—Sb1—Cl3	93.99 (4)	N2—C7—C8	126.0 (3)
Cl4—Sb1—Cl3	85.94 (4)	N1—C7—C8	125.2 (3)
Cl1—Sb1—Cl2	89.66 (4)	C9—C8—C12	118.3 (3)
Cl4—Sb1—Cl2	89.38 (4)	C9—C8—C7	119.8 (3)
Cl3—Sb1—Cl2	173.98 (3)	C12—C8—C7	121.8 (3)
Cl1—Sb1—Cl5	82.62 (3)	N3—C9—C8	119.0 (3)
Cl4—Sb1—Cl5	167.42 (4)	N3—C9—H9A	120.5
Cl3—Sb1—Cl5	85.76 (4)	C8—C9—H9A	120.5
Cl2—Sb1—Cl5	99.46 (4)	C11—N3—C9	123.5 (3)
C2—C1—N1	131.6 (3)	C11—N3—H3B	118.2
C2—C1—C6	122.4 (3)	C9—N3—H3B	118.2
N1—C1—C6	106.0 (3)	N3—C11—C10	119.7 (3)
C3—C2—C1	116.0 (3)	N3—C11—H11A	120.2
C3—C2—H2A	122.0	C10—C11—H11A	120.2
C1—C2—H2A	122.0	C10—C12—C8	120.6 (3)
C2—C3—C4	121.5 (4)	C10—C12—H12A	119.7
C2—C3—H3A	119.3	C8—C12—H12A	119.7
C4—C3—H3A	119.3	C7—N1—C1	109.4 (3)
C5—C4—C3	122.4 (4)	C7—N1—H1A	125.3
C5—C4—H4A	118.8	C1—N1—H1A	125.3
C3—C4—H4A	118.8	C7—N2—C6	109.5 (3)
C4—C5—C6	116.2 (3)	C7—N2—H2B	125.3
C4—C5—H5A	121.9	C6—N2—H2B	125.3
C6—C5—H5A	121.9	C11—C10—C12	118.9 (4)
N2—C6—C5	132.3 (3)	C11—C10—H10A	120.5
N2—C6—C1	106.3 (3)	C12—C10—H10A	120.5
C5—C6—C1	121.4 (3)	H1WA—O1W—H1WB	117.8

### Hydrogen-bond geometry (Å, °)

**Table:**

*D*—H···*A*	*D*—H	H···*A*	*D*···*A*	*D*—H···*A*
N1—H1A···O1W	0.86	1.82	2.652 (4)	161
N3—H3B···Cl5^i^	0.86	2.28	3.056 (3)	150
N2—H2B···Cl2^ii^	0.86	2.48	3.179 (3)	139
O1W—H1WA···Cl2^iii^	0.85	2.35	3.197 (3)	172
O1W—H1WB···Cl3^iv^	0.85	2.35	3.198 (3)	174

Symmetry codes: (i) *x*, *y*, *z*+1; (ii) −*x*+2, −*y*+1, −*z*+1; (iii) *x*−1, *y*, *z*; (iv) *x*−1, −*y*+1/2, *z*+1/2.

## References

[bb1] Demirayak, S., Abu Mohsen, U. & Karaburun, A. C. (2002). *Eur. J. Med. Chem.* **37**, 255–260. 10.1016/s0223-5234(01)01313-7 11900869

[bb2] Minoura, H., Takeshita, S., Ita, M., Hirosumi, J., Mabuchi, M., Kawamura, I., Nakajima, S., Nakayama, O., Kayakiri, H., Oku, T., Ohkubo-Suzuki, A., Fukagawa, M., Kojo, H., Hanioka, K., Yamasaki, N., Imoto, T., Kobayashi, Y. & Mutoh, S. (2004). *Eur. J. Pharmacol.* **494**, 273–281. 10.1016/j.ejphar.2004.04.038 15212984

[bb3] Pawar, N. S., Dalal, D. S., Shimpi, S. R. & Mahulikar, P. P. (2004). *Eur. J. Pharmacol. Sci.* **21**, 115–118. 10.1016/j.ejps.2003.09.001 14757482

[bb4] Rigaku (2005). *CrystalClear* Rigaku Corporation, Tokyo, Japan.

[bb5] Sheldrick, G. M. (2008). *Acta Cryst.* A**64**, 112–122. 10.1107/S0108767307043930 18156677

